# Hybrid Fiber Optic Cable for Strain Profiling and Crack Growth Measurement in Rock, Cement, and Brittle Installation Media

**DOI:** 10.3390/s22249685

**Published:** 2022-12-10

**Authors:** Samuel Nowak, Taghi Sherizadeh, Mina Esmaeelpour, Dogukan Guner, Kutay E. Karadeniz

**Affiliations:** 1 Department of Mining and Explosives Engineering, Missouri University of Science and Technology, Rolla, MO 65409, USA; 2 Department of Electrical and Computer Engineering, Missouri University of Science and Technology, Rolla, MO 65409, USA

**Keywords:** BOTDR, distributed fiber sensing, concrete, brittle, crack detection, structural health monitoring

## Abstract

Brillouin scattering-based distributed fiber optic sensing (DFOS) technologies such as Brillouin optical time domain reflectometry (BOTDR) and Brillouin optical time domain analysis (BOTDA) have broad applicability for the long term and real-time monitoring of large concrete structures, underground mine excavations, pit slopes, and deep subsurface wellbores. When installed in brittle media, however, the meter scale spatial resolution of the BOTDR/A technology prohibits the detection or measurement of highly localized deformations, such as those which form at or along cracks, faults, and other discontinuities. This work presents a novel hybrid fiber optic cable with the ability to self-anchor to any brittle installation media without the need for manual installation along fixed interval points. Laboratory scale testing demonstrates the ability of the hybrid fiber optic cable to measure strains across highly localized deformation zones in both tension and shear. In addition, results show the applicability of the developed technology for strain monitoring in high displacement environments. Linear relationships are proposed for use in estimating the displacement magnitude along discontinuities in brittle media from strain signals collected from the hybrid fiber optic cable. The hybrid fiber optic cable has broad potential applications, such as geomechanical monitoring in underground mines, surface pits, large civil infrastructure projects, and deep subsurface wellbores. The benefits of fiber optic sensing, such as the intrinsic safety of the sensors, the long sensing range, and real time capabilities make this a compelling technique for long term structural health monitoring (SHM) in a wide range of industrial and civil applications.

## 1. Introduction

The use of distributed fiber optic sensing (DFOS) for strain measurements has received an increasing amount of attention for the structural health monitoring (SHM) of large infrastructure projects [1], as well as in deep unconventional well bores [2], tunnels [3], and other underground spaces [4].

Distributed fiber optic sensors are sensitive to linear perturbations at every point along their length, theoretically allowing them to replace thousands of point sensors over kilometer-scale ranges. These attributes make DFOS ideal for monitoring large areas or great distances [5]. Distributed sensing can be accomplished with various methods, among which, Brillouin-based methods have found wide applicability in industrial settings.

Researchers have demonstrated the use of Brillouin optical time domain reflectometry (BOTDR)-based distributed sensing in laboratory and field experiments to monitor underground structures, which included an instrumented rock bolt, shotcrete deformation monitoring, and a displacement sensor for underground mine tunnels, which was later tested at an active mine [3]. This technology was also used to monitor roof strata in a coal mine [6]. The use of DFOS to monitor geologic displacements via installation within a brittle installation media, such as in a grouted borehole, however, requires further investigation; highly localized strains in the optical fiber may go undetected due to the limitations in the spatial resolution of the interrogation system, an effect which was demonstrated experimentally by Madjdabadi (2016) [7].

**Figure 1:**
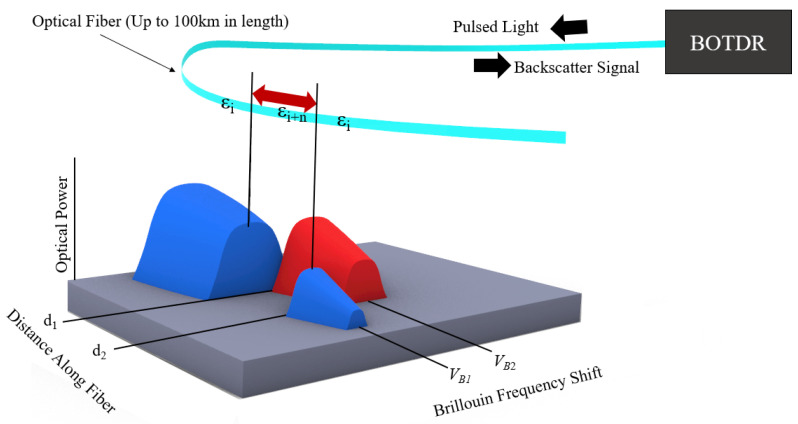
BOTDR measurement principle.

In DFOS, the optical fiber itself is the sensor; thus, the packaging and installation of this sensor within or on the structure to be monitored or measured is critical for the measurement accuracy and interpretability of the received signal. To understand the impact of packaging and installation on the measurement accuracy of BOTDR measurements, we must first consider the Brillouin DFOS measurement principle. A light source is used to launch a beam of light into an optical fiber, resulting in Brillouin backscattering, the frequency of which is dependent on the longitudinal strain along the optical fiber [8]. The position of a strained area along the length of the optical fiber is determined by the time interval between launching a pulse of light from a single end of the optical fiber and receiving the Brillouin backscatter signal [9]. The pulse width from the light source utilized by the BOTDR interrogation system determines the spatial resolution of the measurement, meaning that all strain measurements are averages of the measured strain values within “windows” with a width equal to the spatial resolution. The spatial step determines the distance between the center of each measurement window (Figure 1). Note that when the distance between two locations along the optical fiber trace, d_1_ and d_2_, is far less than that of the spatial resolution of the interrogation system, a lower apparent strain value will be reported due to the averaging process of strain values within the spatial resolution measurement window. This limitation hinders the use of BOTDR for SHM in environments where the optical sensing fiber is likely to be exposed to highly localized strains (those which occur over a length of optical fiber much less than the spatial resolution). Examples of these environments include any application which utilizes a brittle installation media, such as concrete, where displacements occur along cracks or joints.

There are currently two widely accepted methods for installing a fiber optic cable on or within a structure for monitoring: overall bonding, and point fixation. Overall bonding is the process of totally securing a fiber optic cable to the structure for monitoring, while point fixation is the process of securing the fiber optic cable to the structure in points along the cable. Installing fiber optic cables via the point fixation method allows for the selection of fixation intervals, which can be designed to accommodate the spatial resolution of the interrogation system and allow for the measurement of strains over the point fixation interval. The point fixation method also aids in the prevention of cable failure due to the elimination of highly localized strains upon the fragile optical fiber. When using the overall bonding method, the general physical structure of the fiber optic cable layers control the transfer of strain from the environment to the optical fiber core [10]. Specialized fiber optic cables for strain sensing are commercially available and are designed for a wide range of applications, many of which are designed to limit the slippage between cable layers (optical fiber, buffer, strength member, jacket) [11]. The strain transfer between specialty optical strain sensing fibers and the environment when using the full bonding method relies heavily on the bond strength between the jacket material and the surrounding media [12]. Work has been done to characterize the strain transfer/loss at the substrate interfaces using fully bonded fiber optic cables [13]. Existing strain transfer models, however, do not take strain localization into account [14], which becomes critical when the substrate or installation media is prone to cracking. Overall bonded cables are at a high risk of failure when installed in brittle media where highly localized strains are likely to occur during cracking of the media, thus should be used for applications where cracking is not likely to occur [12]. In addition, the meter-scale spatial resolution of most BOTDR interrogators makes crack width measurement difficult or impossible when using overall bonded cables [15]. Several researchers have developed novel methods to integrate optical fibers within structures, or to develop fiber optic cables for specific strain sensing tasks. For example, optical fibers were incorporated into copper wire windings for transformer deformation monitoring [16]. Micro-anchoring of fiber optic cables has been successfully demonstrated for strain profiling in soils by several authors. Micro-anchors are elements which are attached directly to the jacket of the fiber optic cable in order to enhance the coupling of the fiber optic cable to the surrounding media. Micro-anchored fiber optic cables have been used in laboratory scale studies [17,18] and full-scale field applications for monitoring soils during resource extraction [19].

Laboratory scale research has been conducted by Madjabadi (2012, 2016a, 2016b) towards the development of a novel fiber optic cable design for accurate strain measurements in boreholes drilled in rock and filled with brittle media. The experiments compared the performance of various optical fiber installation techniques (point-fixation, overall bonded, and micro-anchored with intentionally de-bonded areas between anchor points) for strain measurement along progressively widening cracks in a concrete beam [7,20,21]. The micro-anchored cable with intentionally de-bonded areas proved to be the best design; however, the de-bonding was achieved through the application of a gel to the outside of the cable and had little commercial viability.

The use of interval bonding, such as the anchored and intentionally de-bonded cable proposed by [7,20,21], permits the easy interpretation of DOFS data, in that the strain can be directly equated to displacement if the interval selected is sufficiently greater than the spatial resolution of the interrogation system, and the bonding of the optical fiber to the installation media is sufficiently strong such that slippage does not occur. It is evident that there is a need for a commercially viable and practically feasible sensing cable that can combine the advantages of point-fixation installations (minimal slippage, known gauge length, easy interpretation) with the straightforward installation process of overall bonded cables (cemented in place in boreholes or large structures).

## 2. Materials and Methods

### 2.1. Hybrid Fiber Optic Cable for Strain Sensing in Brittle Media

This research presents a novel sensing cable design for use in brittle installation media where physical point-fixation is not possible due to accessibility limitations, such as within deep boreholes. The presented sensing cable is designed for the detection of crack development, as well as shear and tensile displacement along highly localized discontinuities such as rock faults, shear zones, and structural cracks in concrete. The developed technology is a hybrid fiber optic cable which consists of a tight buffered optical fiber which is bonded to the installation media (cement, grout) at intervals greater than the spatial resolution of the interrogation system. At all other points along the length of the fiber optic cable, a loose strength member is used to insulate the optical fiber core from highly localized strains. Micro-anchors are used to mate the interval bonded sections of the hybrid fiber optic cable to the installation media. The result is an optical fiber cable which, when strained, produces a constant strained length. Selecting a bonding and de-bonding interval (gauge length) suitable for the spatial resolution of the interrogation system (by keeping the distance between anchoring points greater than the spatial resolution), permits the measurement of cracking in brittle media, as well as the calculation of displacement along cracks from strain measurements (due to the known initial length). The hybrid fiber optic cable is a two-component sensing system. The interval bond secures the optical fiber to the micro-anchor, and the micro-anchor securing the optical fiber to the installation media (grout/cement) and preventing slippage when strained.

**Figure 2:**
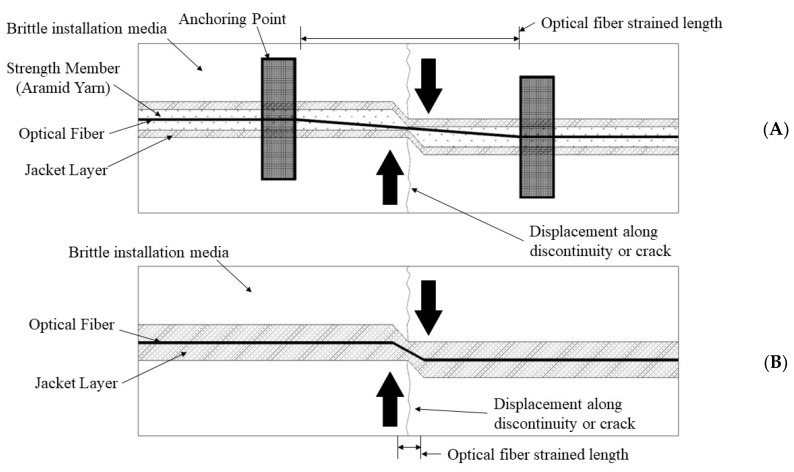
Schematic of the hybrid fiber optic cable for strain sensing in brittle installation media. (**A**) The hybrid fiber optic cable. Note that the optical fiber is insulated from highly localized strains and a known strained length is produced between anchoring points. (**B**) A tight jacketed optical fiber which is fixed to the installation media using the overall bonding technique. Note that a highly localized and thus undetectable strain is produced when displacement along the crack occurs.

An illustration of the principle behind the hybrid fiber optic cable for strain sensing in brittle media is presented in Figure 2.

This method has been proposed by several researchers in the distributed fiber optic sensing space, namely Madjdabadi (2016a, 2016b, 2012) [7,20,21], as well as Tang (2015), who proposed a long gauge distributed fiber sensor for monitoring in concrete beams [22]. Most recently an interval gauged optical fiber was proposed by Han (2021) [18]. However, to the authors’ knowledge, applications of this technology are highly limited, and no practical method for constructing a hybrid fiber optic cable for strain sensing in brittle media with the ability to self-anchor has been proposed. Such a cable, which is designed for long range practical installation, would provide valuable SHM capabilities to a wide range of industrial and civil applications, namely for monitoring subsurface boreholes, mine excavations, rock slopes, and large concrete structures such as dams.

### 2.2. Interpretation of Strain Measurements from the Hybrid Fiber Optic Cable

In order to properly interpret the data collected by DOFS using the developed hybrid fiber optic cable, a connection must be formed between the measured signal (strain on the fiber core between anchoring points) and the displacement or deformation which occurs in the rock mass which the DOFS is installed within. While the use of successive relative strain measurements alone are useful for the localization of a moving zone of rock mass and can be used to identify immediate safety concerns, the ability to estimate the magnitude of deformation, as well as the type (shearing or tension dominant), is the level of data which is required for a deeper understanding of the rock mass response to a particular excavation and could prove useful for the validation/calibration of geomechanical models and support designs.

**Figure 3:**
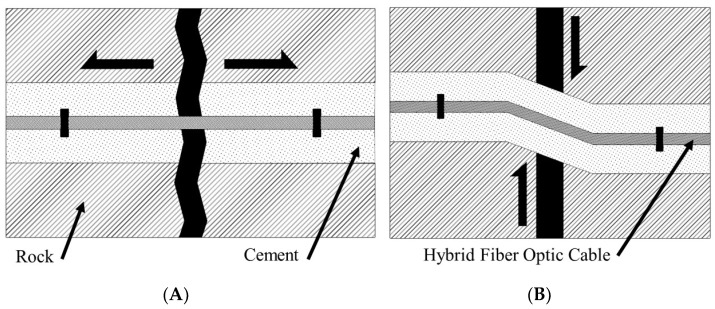
(**A**) Tensile and (**B**) shear displacement along a discontinuity across and grouted borehole with installed hybrid fiber optic cable.

Consider the following example: A DOFS installed with the hybrid fiber optic cable in a grouted borehole within a rock mass (Figure 3). The goal of monitoring in this scenario is to localize the zone of deformation (central black crack in Figure 3A,B) and quantify the amount of displacement which has occurred in the rock mass along the discontinuity.

**Figure 4:**
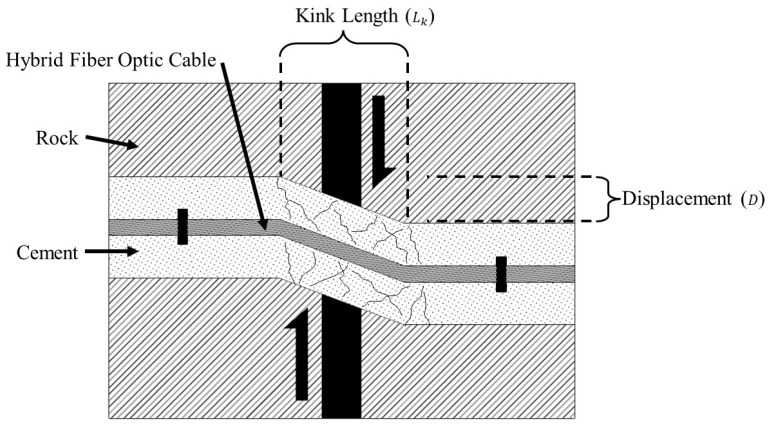
Development of a deformation zone within a cemented borehole in response to shear displacement along a discontinuity, characterized by the kink length. Note: while the kink length can be used to describe the total strain applied to the optical fiber, the strained length of the optical fiber is determined by the gauge length between anchoring points.

In both cases (Figure 3A,B), the strained length of the optical fiber is identical and can be taken as the gauge length or the distance between the two anchoring points (black squares in Figure 3). Both cases will produce axial strains on the optical fiber between the anchoring points, which can be measured with the same accuracy, as both strained lengths are of sufficient length compared to the spatial resolution of the interrogation system. A displacement along the discontinuity in Figure 3A (pure tensile separation of the left and right blocks) will result in a strain along the optical fiber between the two anchoring points, for which the displacement can easily be calculated using the standard strain equation:

(1) $$\varepsilon =\frac{\Delta L}{L_{G}}$$

where ε is the measured strain, $L_{G}$, is the gauge length (the distance between anchoring points), and ΔL is the displacement that has occurred along the discontinuity. In the second case (Figure 3B), however, the strain applied to the fiber is a function of the zone of deformation which occurs as a result of the cement installation media cracking in response to the shear displacement. This zone of crushed or damaged cement is known as the “kink length”, Figure 4.

The strain placed upon the optical fiber for the case illustrated in Figure 4 can be described in terms of the kink length as follows:

(2) $$\varepsilon =\frac{\sqrt{D^{2}+L_{k}{}^{2}}-L_{k}}{L_{G}}$$

where D is the displacement that has occurred along the discontinuity, $L_{k}$ is the kink length, and $L_{G}$ is the gauge length. With this understanding, we can assume that if the kink length which develops in the cement installation media can be well understood, then the displacement magnitude can be calculated from the measured strain interval for a given location along the path of the wellbore. This assumption, however, does require some knowledge of the conditions and expected displacement types associated with the discontinuities at the monitoring site; for example, strains measured from a DOFS installed vertically across sedimentary bedding planes may be reasonably understood to be a result of bedding separation, and thus the measured strain can be directly related to the displacement which has occurred using Equation (1), but strains measured from a DOFS installed perpendicular to a wedge failure plane will require knowledge of the kink length which is likely to form, thus Equation (2) would be used to calculate the displacement which has taken place along the failure plane.

### 2.3. Fabrication of the Hybrid Fiber Optic Cable

A hybrid fiber optic cable for distributed strain sensing [23] in brittle mediums is presented which encompasses both the rationale for DOFS strain sensing in grouted borehole put forth in the previous section, and the operational considerations of fiber optic cable manufacturing and installation at active industrial/infrastructure sites. The first step in the design of the hybrid sensing cable is the selection of a commercially available fiber optic cable from which the modifications are built. The cable selected was a readily available, indoor–outdoor, plenum-rated distribution cable with an aramid yarn strength member, two single-mode tight-buffered optical fibers, and a flame-retardant polyvinyl chloride (PVC) outer jacket, with a nominal outer diameter of 4.4 mm. This cable was chosen as a low-cost option for prototyping/laboratory scale testing reasons; however, the hybrid cable design is adaptable to a wide range of available cables. For field applications, a cable with a thicker jacket layer and more substantial armoring may be required, provided that the optical fiber can be bonded to the external jacket layer. The tight buffer layer around the fiber cores is a commonly used material for strain sensing and prohibits the slippage of the fiber core within the buffer material. Due to the impracticality of laboratory-scale testing for long concrete specimens, an initial gauge length (the distance between the anchoring points) of 0.3 m was selected.

**Figure 5:**
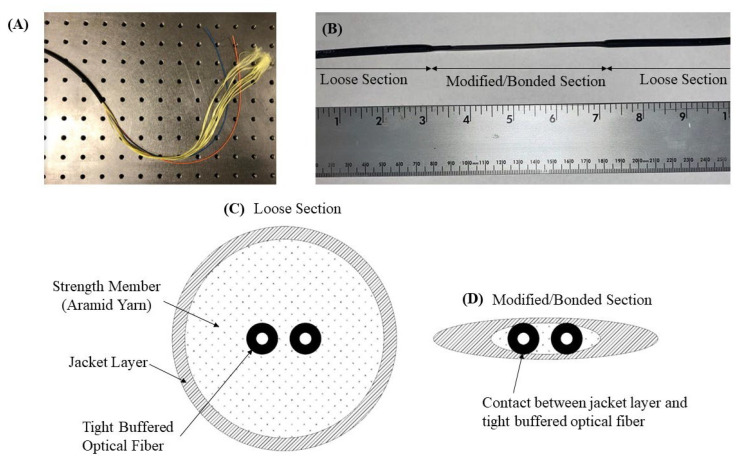
(**A**) The commercially available standard fiber optic cable (note loose contact between optical fibers (orange and blue) and jacket material (black) due to the presence of the aramid yarn (yellow) strength member), (**B**) The profile of the modified hybrid cable showing an interval bonded section where the jacket has been heated and pressed to contact the tight buffered optical fibers, (**C**) A cross section of the standard optical fiber cable and loose section of the hybrid fiber optic cable, (**D**) A cross section of the interval bonded section of the hybrid fiber optic cable (note the contact between the jacket material and the tight buffered optical fibers).

In order to secure the tight-buffered optical fiber cores to the outer jacket layer at increments equal to the gauge length (0.3 m), a heating element was used to heat a 10 cm section of the jacket until pliable. The heated area was then pressed flat, and the melted jacket section allowed to cool. During the melting process, the heated jacket displaces the aramid yarn strength member and comes into direct contact with the tight-buffered optical fibers, Figure 5. The heating process is repeated along the length of the sensing fibers on 0.3 m centers. The result is a tight-buffered optical fiber that is bonded to the outer jacket material at a known increment, while the remaining jacket layer and aramid yarn strength member act as de-bonding agents between the optical fiber and the cement installation media. While the pullout strength of the bonded sections has yet to be thoroughly tested, it is estimated to fall in the range of 60 and 100 Newtons. The addition of the bonded sections to the fiber cable produced no noticeable degradation of the optical signal nor strength/durability of the cable assembly itself, however, the long-term effects of this modification are yet to be studied. Factors such as water aging have been shown to increase fiber attenuation [24], which is a problem which may be exacerbated by the addition of the interval bonded sections. This manufacturing technique is one of many which could be used to secure the optical fiber to the jacket in interval sections and is sufficient for short term installations and laboratory experiments.

**Figure 6:**
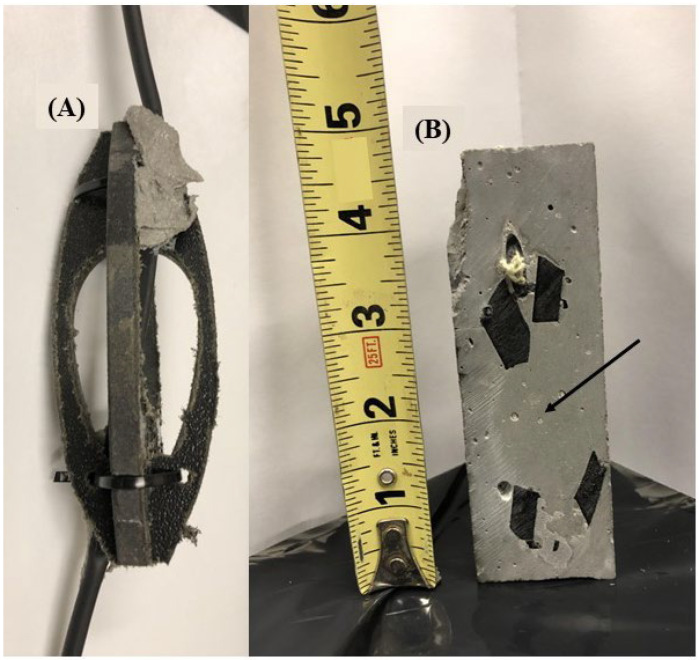
(**A**) The anchoring point attached to the fiber cable at a bonded section using cementitious epoxy resin. (**B**) A cross section of the anchoring point cut from a cement cylinder with an installed anchored fiber. Note the infiltration of grout/cement to the central area of the anchoring point (denoted by black arrow).

The optical fiber must be bonded to the grout/cement within the borehole at an increment equal to the pre-determined gauge length. To accomplish this, an anchoring point (micro-anchor) was designed to attach directly to the fiber optic cable at the bonded sections (Figure 6A). The anchoring point is constructed from high-density polyethylene (HDPE) and can be manufactured in large quantities using a high-pressure waterjet or other standard manufacturing technique. The anchoring point comprised two interlocking parts which are assembled around the fiber optic cable and fixed in place using a cementitious epoxy resin. Cable ties are used to secure the fiber to the anchoring point while the cementitious epoxy resin is applied/cures. The design of the anchoring points, having an open interior space, allows for the infiltration of grout/cement during installation within the borehole, as evidenced by a cross section (Figure 6B) which was cut from a cylindrical cement specimen with an installed anchored fiber optic cable. The anchoring point is designed with a sloping profile to limit catching on the borehole wall during installation.

The hybrid fiber optic cable, having both the bonded sections and anchoring points attached along the length of the cable at an increment equal to the gauge length, can be thought of as a series of high precision, independent strain gauges, end to end. The resulting assembly has the functionality of a multi-point borehole extensometer but lacks the limitations associated with such devices, such as the limited number of points and the susceptibility to electromagnetic interference or water corrosion.

### 2.4. Distributed Fiber Optic Sensing System

A BOTDA fiber optic interrogation system was utilized in this study. The following parameters (Table 1) were used for each of the presented experiments:

### 2.5. Comparison of Commercially Available Fiber Optic Cables to Highly Localized Strains

A large number of fiber optic cables are commercially available for use in distributed strain sensing. Many cables developed for use with the overall bonding method utilize tight buffered optical fibers which are fully bonded to the jacket material, while some utilize various shapes or forms (such as flat shapes or adhesive tapes which maximize the contact area) to allow for a better contact between the optical fiber and the structure to be monitored. Fiber optic cables installed within a grouted borehole may be subject to highly localized strains when cracks develop in the grout. To simulate this event for fiber optic cables installed via the point-fixation method and the overall bonded method on or in a brittle media, a standard elongation test was performed. Several commercially available cables were tested to provide a comparison. The following comparison is by no means exhaustive; however, several common sensing cable structures were selected to provide a wide range of various cable element-to-optical fiber configurations.

**Figure 7:**
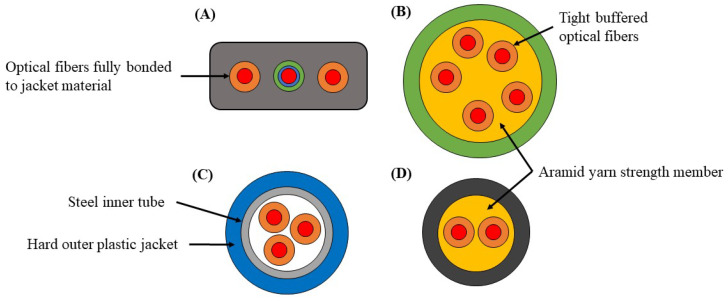
Fiber optic cables selected for comparison under various strain scenarios. (**A**) a strain sensing cable with optical fibers tightly secured to the jacket material (gray). (**B**) a strain sensing cable with optical fibers loosely packed in an aramid yard strength member (yellow) and not in contact with the jacket layer (green). (**C**) A strain sensing cable with a steel inner tube (gray) for additional protection, a hard outer plastic jacket (blue) and three interior tight buffered optical fibers. (**D**) a standard telecommunications cable with a plenum jacket (black) and aramid yarn strength member (yellow), similar in construction to (**B**).

Four commercially available fiber optic cables were selected for the comparison (Figure 7). Three of the cables (Figure 7A–C) are advertised as strain sensing cables, while the fourth (Figure 7D) is a standard telecommunication style cable.

In addition to the four commercially available cables is the hybrid fiber optic cable with modified/bonded sections located at the anchor/clamp points. A bare fiber optic cable was also tested to provide a comparison in measured strain when no jacket or strength member is considered.

**Figure 8:**
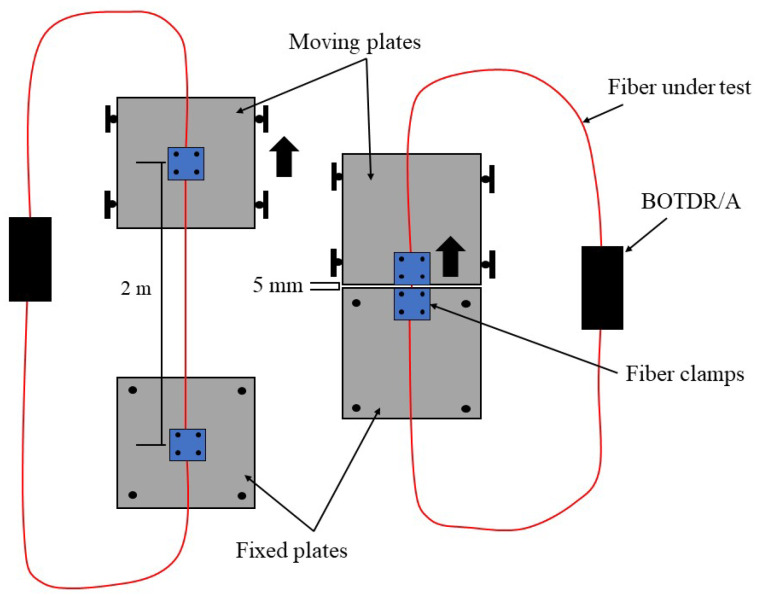
Elongation experimental setup for fiber optic cable comparison.

The various cables were tested in two elongation experiments, similar to those conducted in [7]. The elongation experimental setups were constructed from aluminum plates secured to an optics table (Figure 8). The optical fiber cables were secured to the plates with clamps, which were tightened as much as the fiber optic cable under test would allow without rupturing. One aluminum plate was fixed to the optics table in all directions, while the other (the moving side) was fixed via rollers such that lateral movement was possible. Lateral displacement of the moving side plate was administered with a high precision translation stage, and a dial gauge was used to validate the administered displacement. Two strained lengths were selected, one greater than and one much less than the 1 m spatial resolution of the interrogation system. The larger strained length (2 m) is used to demonstrate a point-fixed fiber optic cable installed over crack, and the smaller strained length (5 mm) is used to demonstrate a fully bonded fiber optic cable installed in brittle media in which a crack forms and expands. A total displacement of 2 mm was applied to each cable in each scenario.

**Figure 9:**
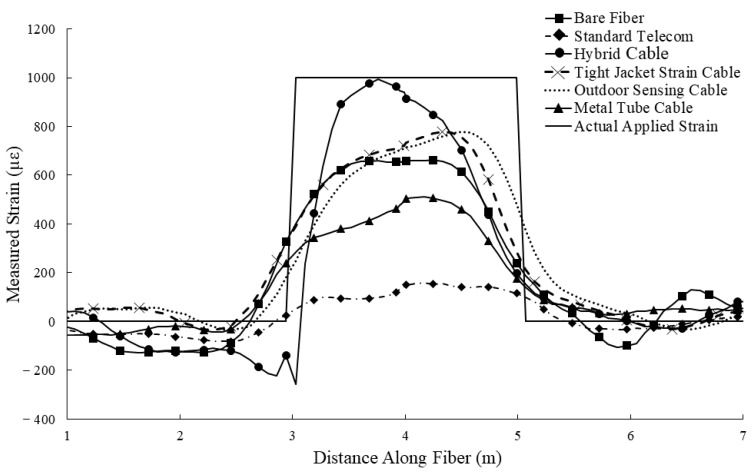
Comparison of various commercially available fiber optic cables in an elongation test, strained length is 2 m.

The results of the elongation experiment for the 2 m strained length are presented in Figure 9. As expected, no discernable signal was produced with any cable for the 5 mm strained length experiment, illustrating the limitations of the full bonding method for distributed sensing using meter order spatial resolutions in brittle media for crack measurement.

The 2 m strained length point fixation elongation experiment serves to demonstrate the effect of cable construction on measured strain signals when fiber cable clamps are used to secure the fiber cable to the structure. The aramid yarn strength member in the standard telecom fiber cable (Figure 7D) likely precluded the ability of the fiber cable clamps to adequately hold the optical fiber in place during the experiment, resulting in slippage and a poor-quality measurement. Cables designed/marketed explicitly for strain sensing (Figure 7A,B) performed on par with the bare optical fiber. The metal tube component of the armored fiber cable (Figure 7C), designed for strain and temperature sensing in harsh environments, also precluded the clamping mechanism on the experimental setup to fully engage the optical fiber, resulting in slippage and poor-quality measurement.

The interval bonded sections of the hybrid fiber optic cable permitted the clamping mechanisms to adequately engage the optical fiber, thus resulting in the highest measurement accuracy of the tested cables. Interval bonding of the jacket material to the optical fiber in only the areas necessary (such as those to be point-fixed within a grouted borehole) provides the benefit of reducing the strain lag between the jacket material and the anchor/clamping mechanism.

### 2.6. Experimental Characterization of Hybrid Fiber Optic Cable in Brittle Media

Displacements along discontinues in brittle materials can occur in tension, shear, or some combination of the two. Two laboratory experiments were conducted to simulate the deformation of a grouted borehole within a structure or rock mass along a discontinuity surface in tension (via increasing the aperture of a pre-existing fracture) and in shear (via an orthogonal displacement to the borehole axis). The intent of the performed experiments is to demonstrate the ability of the developed hybrid cable to detect deformation across highly localized zones (such as those around faults, joints, and cracks), as well as its ability to utilize the measured strain signals to calculate displacement along such structures.

A series of cylindrical cement specimens (0.5 m in total length) were prepared as analogs to a grouted borehole with embedded DOFS. Two specimen types were fabricated, one with a standard optical fiber cable (tight buffered optical fiber, aramid yarn strength member, PVC jacket), and one with the hybrid cable design described in the previous section. The samples were formed using a heavy-duty cardboard tube (7.6 cm inner diameter) with tight-fitting plastic endcaps. Portland cement (class 1) was mixed according to [25]. The fiber cables were centered into each mold, and cement poured around them. The samples were then shaken to release any large air bubbles and allowed to cure for at least one week. All optical fiber cables (both standard and hybrid) were pre-tensioned before the cement was allowed to cure, thus preventing any slack buildup in the cable which could affect the strain profiles. Given the large number of available fiber optic cables, laboratory scale testing of each variety is impractical. For comparison reasons, the results for the hybrid fiber optic cable are presented side-by-side with a standard, non-modified version of the same tight buffered optical fiber cable with PVC jacketing material and aramid yarn strength member. This is by no means an exhaustive comparison but serves to demonstrate the efficacy of the hybrid fiber optic cable compared to a non-modified commercially available cable of the same construction materials.

### 2.7. Experimental Setup for Simulating Tensile Deformation in Grouted Boreholes

**Figure 10:**
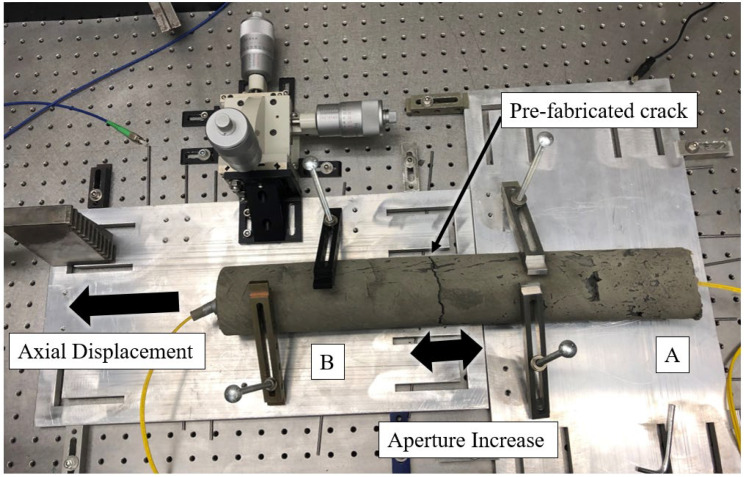
Sliding plates are set up for the administration of axial displacements upon a cylindrical cement beam with embedded fiber optic cables. The test setup is designed to simulate the opening of a discontinuity surface along a borehole trace in which a DOFS is installed within grout or some form of brittle media. A and B denote the fixed and moving sides, respectively.

Many of the SHM systems available to rock mechanics and civil engineers operate by measuring some form of axial displacement (along the long axis of the monitoring instrument). Many monitoring applications require that the instrument be installed orthogonal to a pre-existing discontinuity in order to measure displacement along the discontinuity in the form of fracture widening or aperture increase. To demonstrate the efficacy of the hybrid fiber optic cable design for monitoring fracture development orthogonal to a grouted borehole axis, axial testing was conducted by fixing a cylindrical cement beam with the embedded optical fiber cables upon a set of aluminum plates (Figure 10). The cement beam, which simulates the grouted space of an instrumentation borehole, is pre-cracked in the center of the beam, forming an initial tensile crack from which further displacement can be placed upon the beam in the form of increasing the fracture aperture. One of the aluminum plates remains fixed by clamping to an optical table, while the other side (the moving side) is set upon rollers and constrained in all directions except along the axis of the cement beam. A high precision translation stage is secured to the moving side plate to allow for the incremental administration of tensile displacements upon the sample. A dial gauge is used to measure the total amount of displacement.

**Figure 11:**
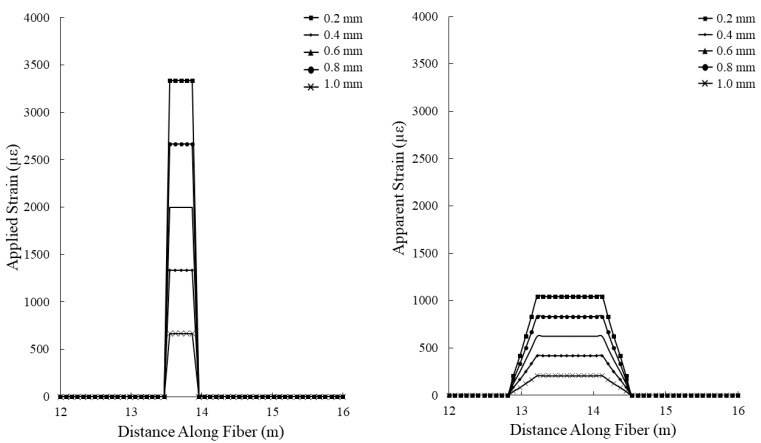
Theoretical applied (**left**) and apparent (**right**) strains incident upon the hybrid optical fiber cable during the tensile deformation experiment. Axial displacements from 0.2 to 1.0 mm. Gauge length is 0.3 m.

The experimental setup for simulating tensile deformation in grouted boreholes is designed to apply an axial displacement to the cement beam in 0.1 mm increments. When considering the 0.3 m gauge length of the hybrid fiber optic cable, the applied strain can be calculated using Equation (1). As the spatial resolution of the interrogation system used in this study is 1m, the expected apparent strain can be calculated by averaging the applied strains over 1 m intervals at increments equal to the spatial step (the distance between centers of spatial-resolution-width measurement windows along the length of the optical fiber) of the interrogation system (0.08 m). When using a gauge length longer than the spatial resolution, the apparent strain is equal to the applied strain. The theoretical applied and apparent strains incident upon optical fiber in the tensile deformation test are presented in Figure 11.

The tensile deformation experimental setup has the added benefit of a long displacement stroke, allowing for the simulation of large tensile deformations, discussed further in the results section of this work.

### 2.8. Experimental Setup for Simulating Shear Deformation in Grouted Boreholes

**Figure 12:**
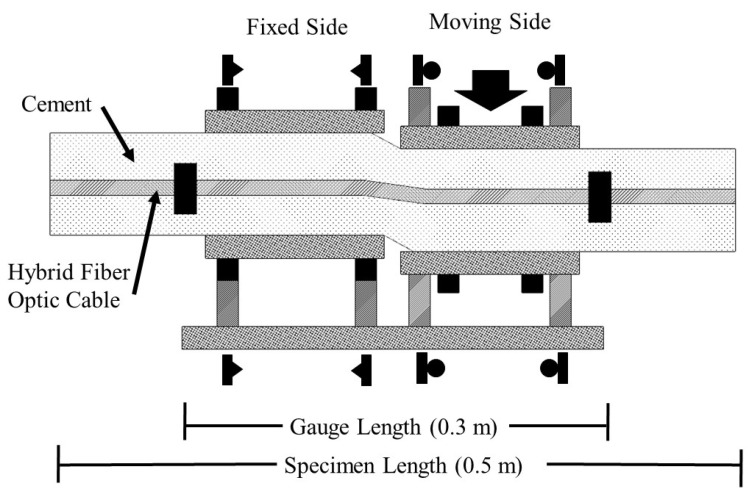
Schematic of the shear displacement fixture.

**Figure 13:**
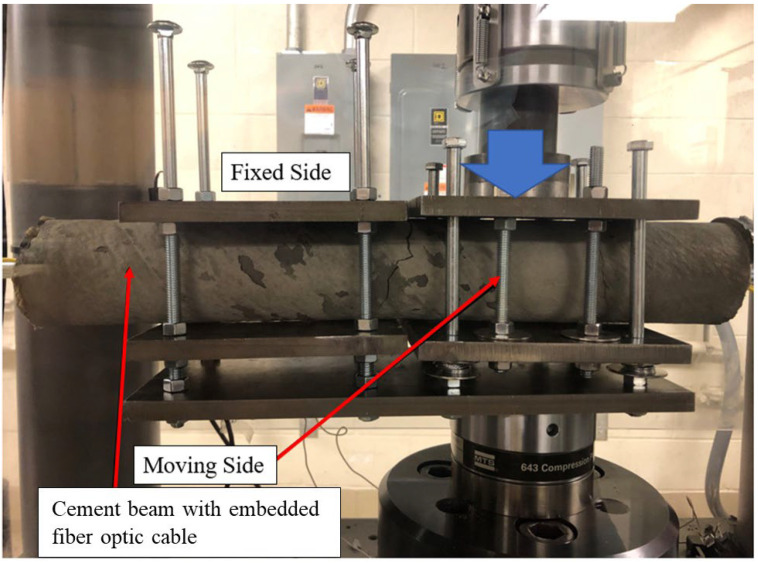
Shear displacement setup with cement sample with embedded hybrid fiber optic cable secured in the hydraulic loading frame.

Installing a DOFS such that only axial strains are incident upon the cable is not always possible, especially in the rock where discontinuity surfaces may intersect a wellbore trace at various angles. To demonstrate the efficacy of the developed hybrid cable in detecting strains due to movement along a discontinuity plane which is orthogonal to the wellbore trace, a specialized fixture was developed to apply a shear displacement upon the 0.5 m cement beam with embedded fiber optic cables using a hydraulic, servo-controlled loading frame. The fixture consists of two sets of 12 mm steel plates which hold the beam (Figure 12 and Figure 13). One set of plates, the fixed side, are fixed in place using threaded carriage bolts. The other set of plates is fixed to the cylindrical cement beam by tightening hex-nuts about four carriage bolts which are used to sandwich the beam between the plates. This set of plates is allowed to slide freely along a set of four upright, un-threaded carriage bolts. Both sets of plates are attached to a common base plate. A load is applied to the top of the moving side plates and bottom of the shared base plate, which results in a shear displacement that is concentrated on the cement beam in the area between the two sets of plates, the gap of which (the aperture) is 5 mm (Figure 13). A total shear displacement of 4 mm can be applied to the sample at 1mm increments, with a displacement rate applied by the loading frame of 0.0001 mm/s.

**Figure 14:**
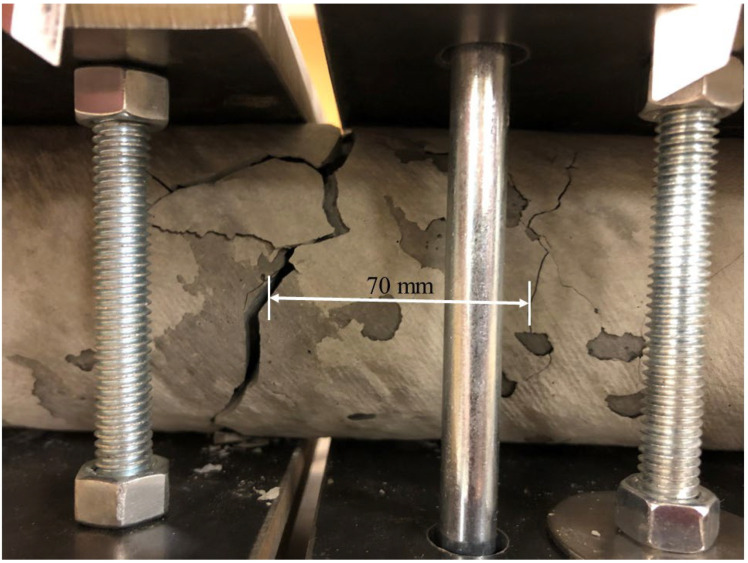
Cracked cement beam after a shear deformation test in which a kink length of 70 mm was developed.

Unlike the tensile deformation experimental setup, the shear deformation setup not designed for use with a pre-cracked cement beam, as sufficient stress can be applied to the cement from the loading frame to crack the beam and administer the shear deformation. During the experiments, a kink length of 70 mm was found to develop in most cases (Figure 14).

**Figure 15:**
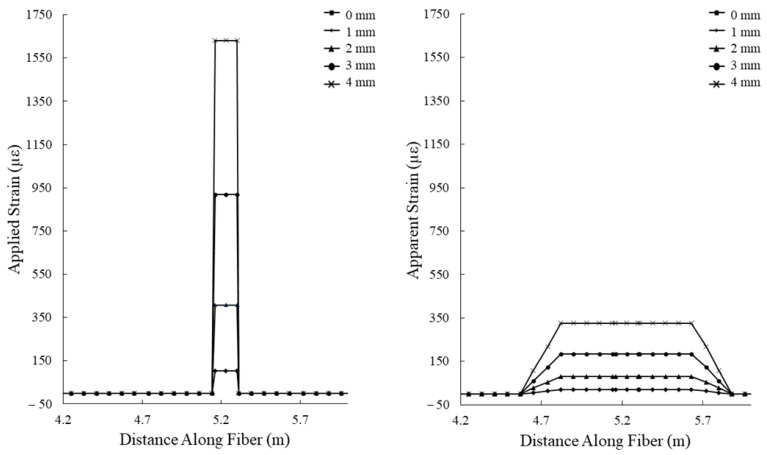
Theoretical applied (**left**) and apparent (**right**) strains incident upon the hybrid optical fiber cable during the shear deformation experiment. Applied shear displacements are from 0.0 to 4.0 mm. Gauge length is 0.3 m, and the kink length noted in the cement beam is 70 mm.

Assuming a constant kink length of 70 mm for the shear deformation test, the theoretical applied and apparent strains can be calculated given the administered shear displacements using Equation (2) (Figure 15). Note that as the use of the hybrid fiber optic cable engages the entire length of the optical fiber between anchoring points in response to highly localized deformations in the cement beam, the applied and apparent strains presented in Figure 14 are only valid for use with the hybrid fiber optic cable, and not for a cable installed using the overall bonding technique.

## 3. Results

### 3.1. Tensile Deformation Test

**Figure 16:**
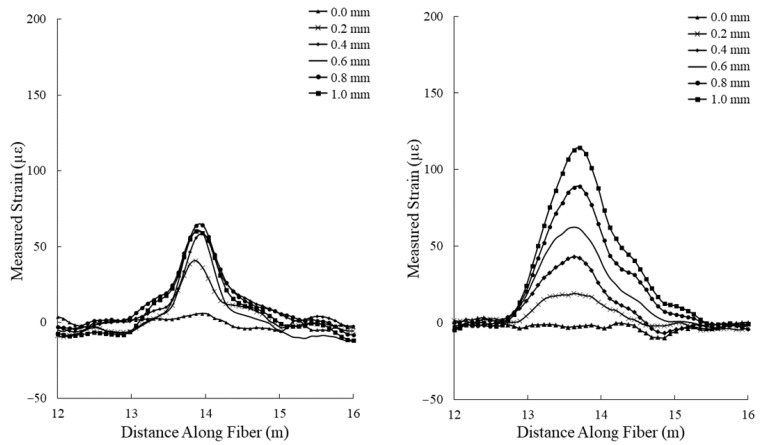
Strain measurements taken for incremental displacement (from 0.0 to 1.0 mm) values during the tensile deformation experiment using a standard fiber optic cable (**left**) and the hybrid fiber optic cable (**right**).

The results of the tensile deformation test are presented in Figure 16. Strain measurements from the standard fiber optic cable reached a maximum of 64 με. This cable was unable to produce a recognizable increasing strain measurement pattern, likely due to the aramid yarn strength member acting as a strain insulator for the optical fiber. Strain measurements made while using the hybrid fiber optic cable resulted in a maximum strain of 114 με, nearly double that of the standard fiber optic cable.

**Figure 17:**
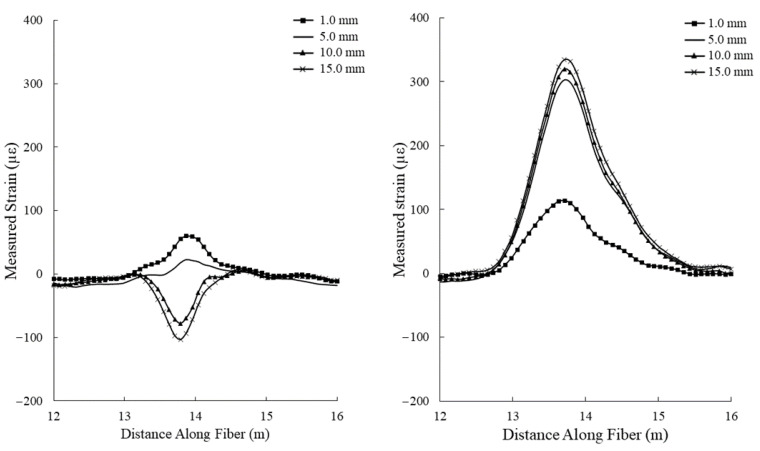
Strain measurements taken for incremental displacement (from 1.0 to 15.0 mm) values during the large tensile deformation experiment using a standard fiber optic cable (**left**) and the hybrid fiber optic cable (**right**). Note that the standard fiber optic cable shows a decreasing strain value at higher displacements, indicating a slippage of the optical fiber within the jacket material.

To analyze the ability of the hybrid fiber optic cable to continue monitoring in high displacement environments, the tensile deformation testing setup was used to administer larger axial displacements to the cement beams with embedded DOFS. Displacements were administered to the cement beams in 5.0 mm increments (Figure 17).

### 3.2. Shear Deformation Test

**Figure 18:**
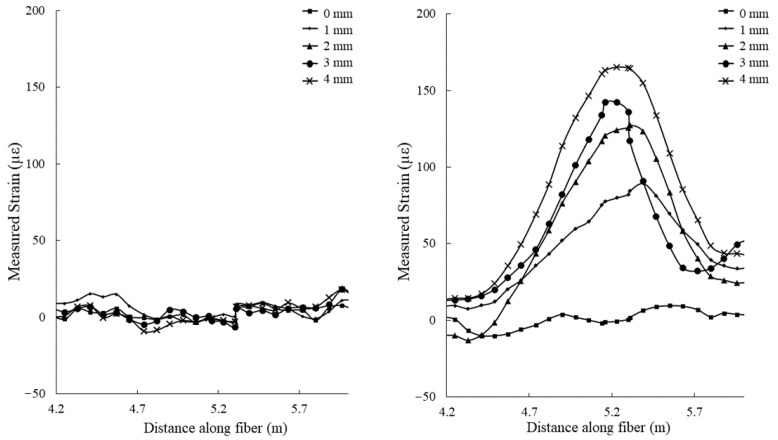
Strain measurements taken for incremental displacement values (from 0 to 4 mm) during the shear deformation experiment using a standard fiber optic cable (**left**) and the hybrid fiber optic cable (**right**).

The results of the shear deformation test are presented in Figure 18. Strain measurements from the standard fiber optic cable reached a maximum of 7 με, which is below the detection limit considering the ± 40 με noise level produced by the interrogation system. This cable was unable to produce a recognizable increasing strain measurement pattern, likely due to the aramid yarn strength member acting as a strain insulator for the optical fiber. Strain measurements made while using the hybrid fiber optic cable resulted in a maximum strain of 165 με, representing a strain signal increase greater than 2000%.

## 4. Discussion

**Figure 19:**
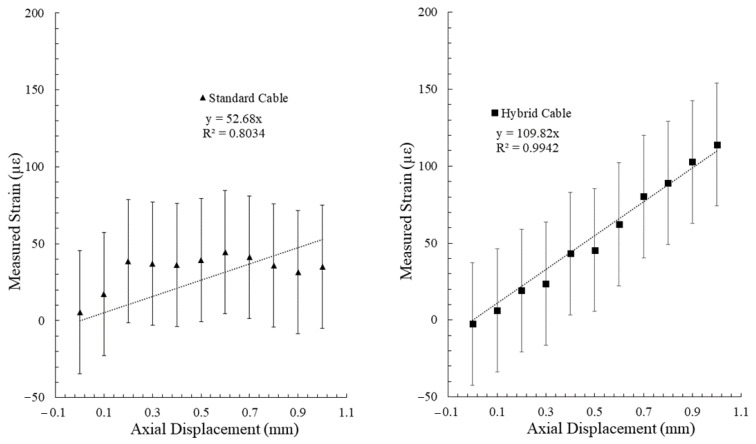
Measured strain verses applied axial displacement for a standard optical fiber cable (**left**) and the developed hybrid fiber optic cable (**right**).

**Figure 20:**
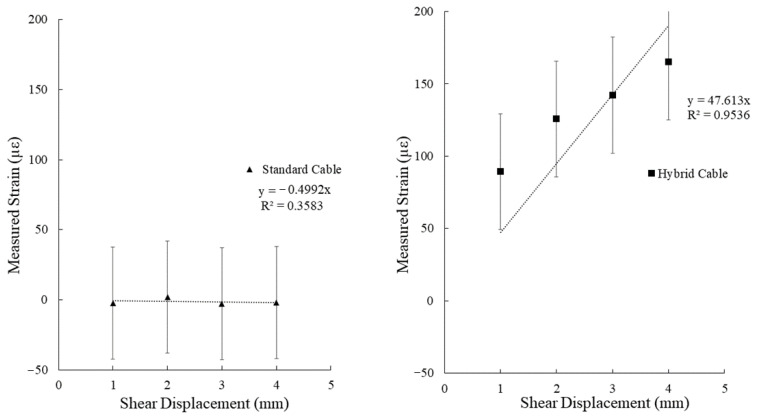
Measured strain verses applied shear displacement for a standard optical fiber cable (**left**) and the developed hybrid fiber optic cable (**right**).

The BOTDA distributed fiber optic interrogation system with the settings utilized produces a static noise level of ± 40 με, thus any displacement estimations utilizing this system will have an error of at least this value, plus any error found within the manufacturing process (namely in the distance between anchoring points). By comparing the maximum measured strain from the optical fiber trace in the area of interest (the center of the cement beam) for each incremental applied displacement, the efficacy of the two cable designs (standard vs. hybrid) can be visualized. The results for the tensile and shear deformation tests are presented in Figure 19 and Figure 20, respectively.

A clear linear trend between the applied displacement and measured strain for the hybrid fiber optic cable is noted for the tensile deformation experiment. It should be noted, however, that this trend does not continue for higher displacement values, indicating a potential bi-linear to polynomial relationship for higher displacement/strain environments. A linear trend was assigned to the hybrid fiber optic cable measurements for the shear deformation experiment. While the ability of the developed shear testing apparatus in delivering high precision displacements under 1mm is still lacking, a near linear trend between displacement and measured strain can still be inferred below 1mm from the collected measurements by extending this relationship.

**Figure 21:**
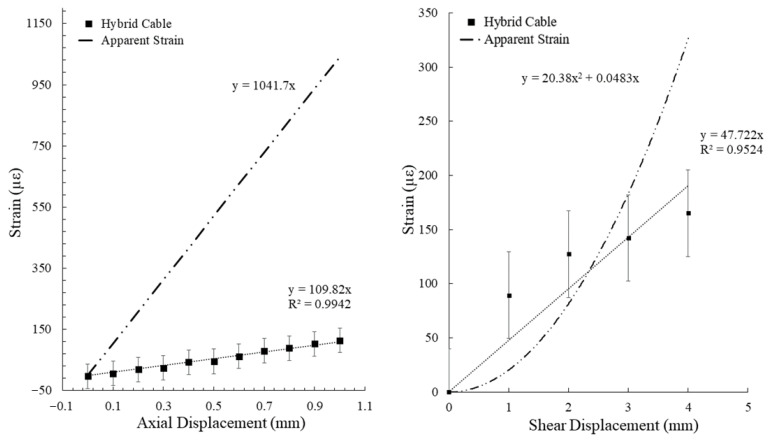
Apparent strain and measured strain when using the hybrid fiber optic cable in the tensile deformation experiment (**left**) and shear deformation experiment (**right**).

A comparison between the measured strain signals using the hybrid fiber optic cable and the expected apparent strain can also be used to assess the efficacy of the sensor in monitoring kinematic deformation in brittle media (Figure 21).

Linear trends in both the apparent and measured strains for the tensile deformation experiment indicate a good agreement between the theoretical strain environment and the sensor, despite the disparity in strain magnitude. The results from the shear deformation experiment indicate a better agreement in strain magnitude between the expected and measured values but a disparity in the overall trend.

**Figure 22:**
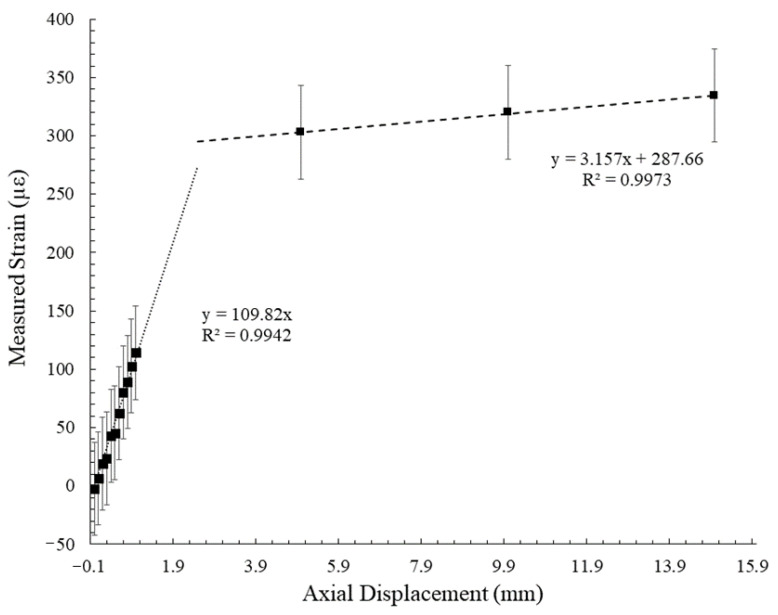
Measured strain values for tensile deformation experiments using the hybrid fiber optic cable. Note the bi-linear relationship between measured strain and displacement in high displacement environments.

The large tensile deformation experiments demonstrate the effectiveness of the hybrid fiber optic cable in measuring crack growth in high displacement environments. Evidence of un-controlled debonding from the standard fiber optic cable can be noted by the decreasing strain signal and eventual large negative strain value after an axial displacement of 15.0 mm was administered (Figure 17). While the hybrid fiber optic cable was able to continue monitoring in a high displacement environment, the relationship between applied displacement and measured strain did not follow the same trend as has been observed in the smaller tensile deformation experiment (Figure 22). A bi-linear trend can be assigned to describe this relationship. It should be noted that the measured strains in the high displacement experiment depart further from the linear apparent strain trend than the small strain experiments, indicating the potential existence of one or more displacement cut-off zones, where the proposed relationships are valid. In addition, the high displacement segment of the bi-linear relationship indicates that measured strain at high displacements while using the hybrid fiber optic cable may be heavily controlled by the plastic yielding of the optical fiber itself.

The proposed linear relationships have been developed via laboratory scale experiments on grouted hybrid fiber optic cables with a single discontinuity that is perpendicular to the cable trace. The strained length of optical fiber for each test was 0.3 m; thus, the relationships between applied displacement and measured strain are expected to be different for strained lengths over the spatial resolution, where the applied strain and apparent strain will be equal at the midpoint of the strained fiber. In a field scale installation, discontinuities at angles other than perpendicular to the optical fiber trace will be encountered. This will require additional information on the formation of kink lengths within the installation media to find the applied strain to the area of interest. Logically, the expected measured strain values for an optical fiber installed along an angled discontinuity may approach somewhere between the two extreme cases (displacements applied at 180° and 90° to the optical fiber trace) presented in this work. This requires some understanding of the installation configuration, and the expected type of movement along the expected discontinuity surface(s). During field installations where temperature variations are expected to occur, a second loose jacketed optical fiber cable insulated from mechanical strains is suggested for deployment alongside the hybrid fiber optic cable so that temperature correction can be accomplished. Based on the underlying basis for the hybrid fiber optic cable design, any jacketing materials, such as steel or other high strength cable materials, can be used to construct a hybrid fiber optic cable, providing that the optical fiber core is secured to the anchoring points and thus the brittle installation media.

In this research, the long-term effects of field conditions (water-aging, optical fiber deterioration) which may result from the addition of the modified/bonded sections have not been studied but are currently being considered. The manufacturing technique utilized in this work to construct the modified/bonded sections (through the use of heating element) is a first attempt, and other manufacturing techniques may be more suitable and more repeatable for long-term distributed fiber optic deployments than those proposed here.

## 5. Conclusions

In this work, a novel hybrid fiber optic cable is proposed to overcome the limitations of distributed fiber optic sensing for crack detection and monitoring in brittle media. The developed cable design combines the measurement accuracy of the point-fixation fiber optic installation method with the practical ease of installation associated with the overall bonding method. Several conclusions can be drawn from this work:

— The hybrid fiber optic cable design and manufacturing process is simple enough to be manufactured on a wide scale, permitting the use of this technique for a wide range of industrial applications.
— The use of the hybrid fiber optic cable ensures that a constant strained length (gauge length between anchoring points) is formed in response to highly localized deformations, such as those found in cracks in brittle media.
— The use of modified/bonded sections has a positive impact in securing the optical fiber to anchor/clamping mechanisms and has been to reduce the effect of strain lag between the cable jacket and optical fiber.
— The gauge length of the hybrid fiber optic cable can be selected and tailored for the application and the limitations of the interrogation system (namely the spatial resolution).
— The hybrid fiber optic cable produced strain signals which were significantly more accurate and more identifiable than a comparable overall bonded fiber optic cable for strain sensing across a highly localized tensile deformation zone and shear deformation zone, respectively.
— Linear trends are proposed to relate the measured strain to applied displacements along highly localized deformation zones in tension and shear.
— The hybrid fiber optic cable was demonstrated to be effective for monitoring in high strain environments, where overall bonded cables may undergo un-controlled de-bonding and report erroneous strain measurements.
— Knowledge of the environment in which the hybrid fiber optic cable is to be installed may be used to improve the estimation of displacements from strain measurements. Such information may aid the user in determining if a strain measurement is expected to have originated from a tensile dominant or shear dominant deformation source.
— Estimates of the kink length which forms in a cement beam under shear dominant deformations are needed for accurate displacement estimations from strain measurements using the hybrid fiber optic cable.

The hybrid fiber optic cable has broad potential applications, such as for geomechanical monitoring in underground mines, surface pits, large civil infrastructure projects, and deep subsurface wellbores. The benefits of fiber optic sensing, such as the intrinsic safety of the sensors, the long sensing range, and real-time capabilities make this a compelling technique for long-term SHM in a wide range of industrial and civil applications.

Further work is needed to assess the efficacy of the sensor in compressional environments where the optical fiber may be relaxed instead of tensioned. In addition, large scale testing should be conducted in the field to assess the practicality of installation at industrial scales, as well as the interpretation of collected data.

## 6. Patents

The hybrid fiber optic cable described in this work is patent-pending technology [23].

## Figures and Tables

**Table 1:** Parameters used for BOTDA distributed strain measurements.

Spatial Resolution	Spatial Step	Averages	Pulse Width	Fiber Strain Coefficient
1 m	0.08 m	60,000	10 ns	18.915 με/MHz

## Data Availability

Not applicable.
